# Infective Endocarditis-Induced Lung Injury Mimicking Acute Vanishing Lung Syndrome

**DOI:** 10.7759/cureus.35454

**Published:** 2023-02-25

**Authors:** Oluwafemi Ajibola, Taiwo O Aremu, Olawale Ajibola, Oluwatosin E Oluwole, Bennett P DeBoisblanc

**Affiliations:** 1 Section of Pulmonary and Critical Care Medicine, Louisiana State University Health Sciences Center, New Orleans, USA; 2 Division of Environmental Health Sciences, School of Public Health, University of Minnesota, Minneapolis, USA; 3 Department of Pediatrics, University of Minnesota, Minneapolis, USA; 4 Department of Pharmaceutical Care & Health Systems, College of Pharmacy, University of Minnesota, Minneapolis, USA; 5 Medicine, American University of St. Vincent School of Medicine, Kingstown, VCT; 6 Division of Epidemiology & Community Health, School of Public Health, University of Minnesota, Minneapolis, USA

**Keywords:** staphylococcus auerus, methicillin resistant staphylococcus aureus (mrsa), pneumothoraces, antibiotics, pneumatoceles, acute vanishing lung syndrome, infective endocarditis

## Abstract

Infective endocarditis is the infection of the endocardial surface of the heart valve. The right-sided endocarditis can be complicated by pulmonary injury. The pulmonary complications of infective endocarditis include pulmonary embolism, empyema, pleural effusion, lung abscess, and, in rare cases, pneumothorax. We present a case of bilateral pneumatoceles mimicking vanishing lung syndrome, a very rare pulmonary complication of right-sided infective endocarditis.

## Introduction

Infective endocarditis (IE) is an infection of the endocardial surface of the heart, mainly caused by *Staphylococcus aureus*, a Gram-positive and non-motile bacterium. Among intravenous drug users, methicillin-resistant *Staphylococcus aureus* (MRSA) is one of the most common causes of IE [1]. The annual incidence of IE ranges from three to seven cases per 100,000 person-years [2]. IE can progress very quickly to present with systemic complications. The pulmonary complications of right-sided IE include pulmonary embolism with infarction, abscess formation, and rarely pneumothorax. Right-sided IE most commonly involves the tricuspid valve and is associated with intravenous drug use or the use of indwelling vascular catheters. Even with appropriate antibiotics and surgery, the clinical resolution of these injuries can mimic vanishing lung syndrome, also referred to as idiopathic giant bullous emphysema, which is a rare pulmonary complication of right-sided IE. We present an unusual case of bilateral pneumatoceles mimicking vanishing lung syndrome.

## Case presentation

**Figure 1:**
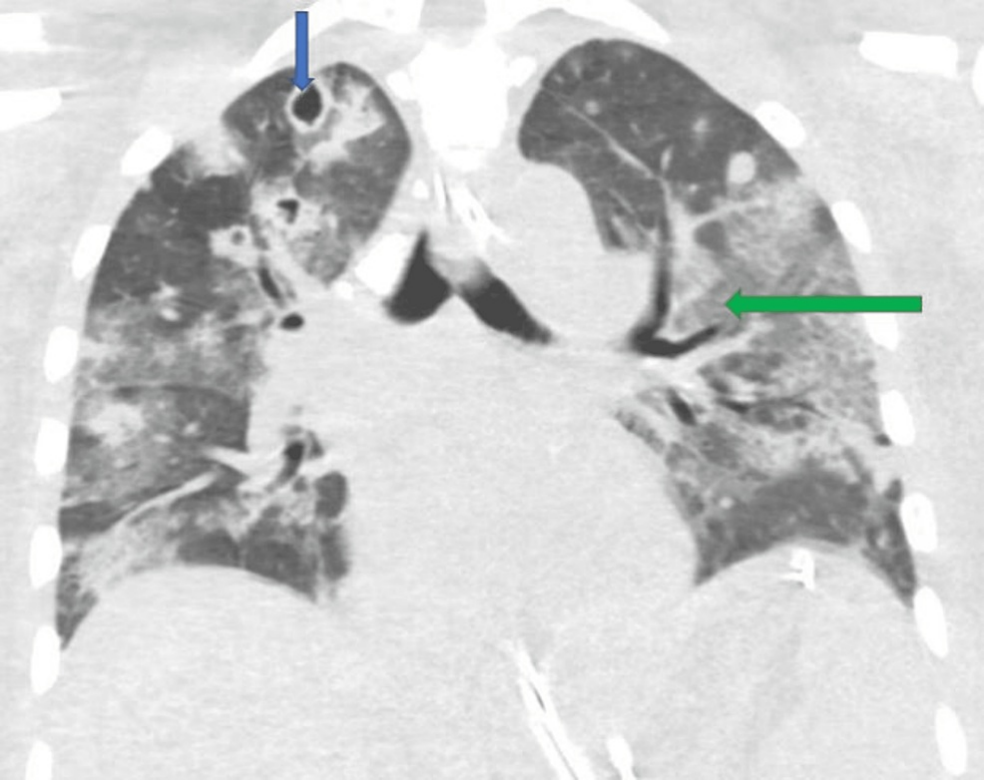
Computed tomography (CT) scan of the chest (day 1) showing multifocal confluent consolidations and cavitary lesions with superimposed diffuse ground-glass opacities. The blue arrow demonstrates a right apical cavitary lesion while the green arrow shows a broad area of ground-glass opacity.

A 31-year-old male with a medical history of intravenous drug use presented with a one-day history of fever, chills, and pleuritic chest pain. His initial vital signs showed blood pressure of 162/72 mmHg, heart rate of 119 beats per minute, temperature of 34.3^o^C, and respiratory rate of 35 cycles per minute. His examination was remarkable for a draining wound to the right upper arm. A computed tomography (CT) scan of the chest showed multifocal confluent consolidations and cavitary lesions with superimposed diffuse ground-glass opacities (Figure 1).

**Figure 2:**
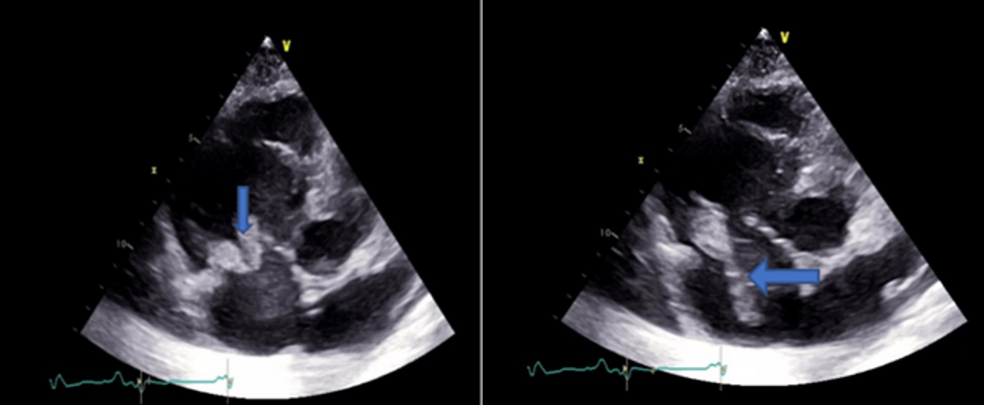
Apical four-chamber echocardiogram. The blue arrows show large and highly mobile vegetations on posterior and septal tricuspid valve leaflets.

He was intubated for respiratory failure and started on broad-spectrum antibiotics including coverage for MRSA. A bedside transthoracic echocardiogram showed large and highly mobile vegetations on posterior and septal tricuspid valve leaflets (Figure 2).

**Figure 3:**
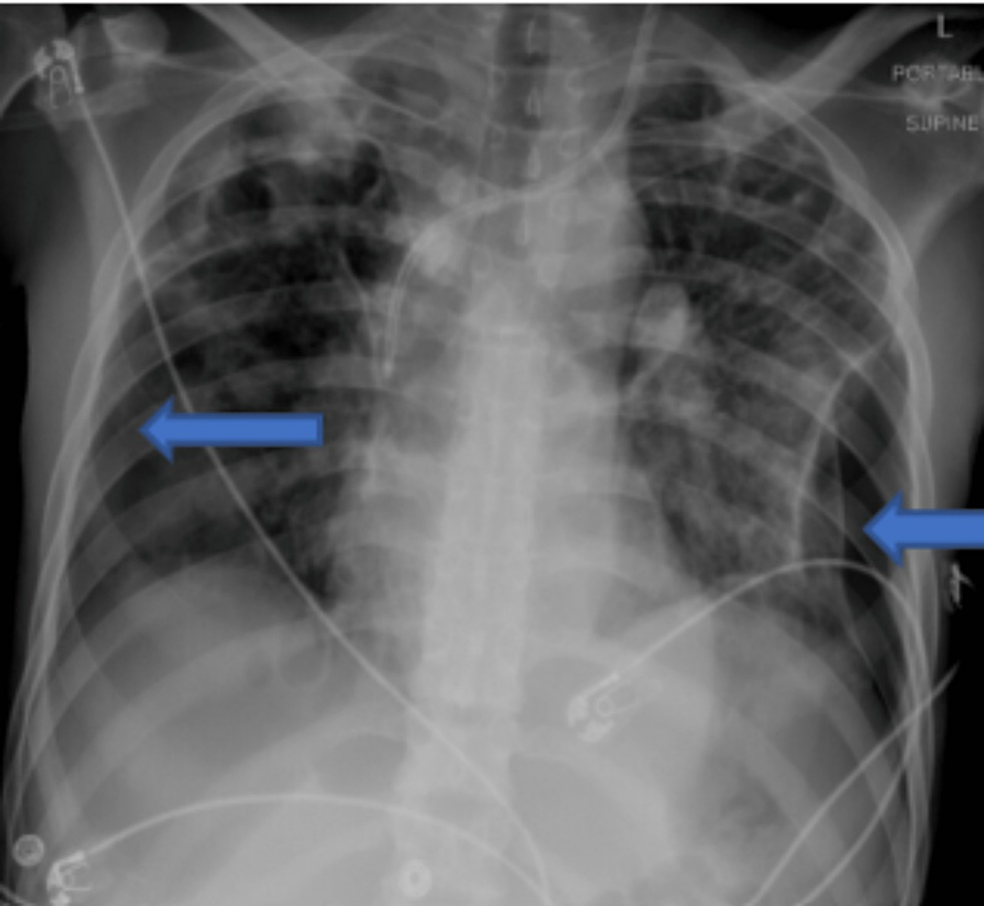
Chest X-ray showing evidence of bilateral pneumothoraces. The blue arrows indicate new left-sided loculated pneumothorax and right-sided pneumothorax.

**Figure 4:**
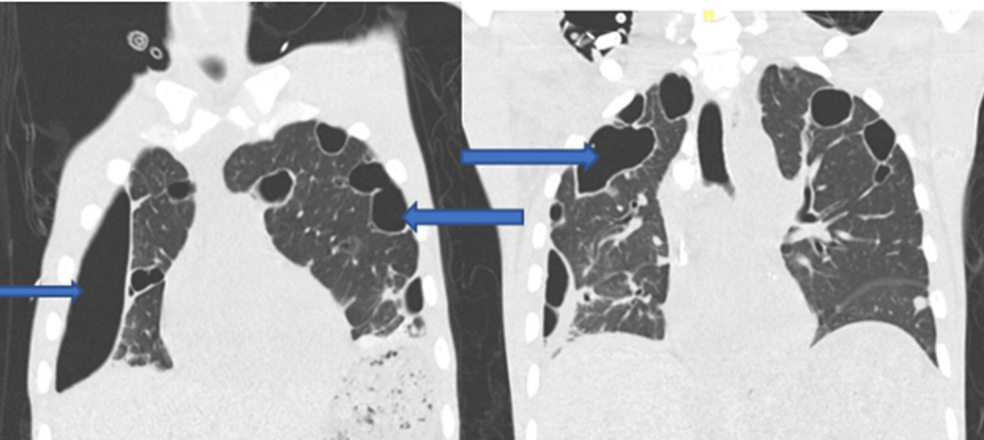
Computed tomography (CT) scan of the chest. The blue arrows identify the loculated right-sided pneumothorax and multiple pneumatoceles.

Blood cultures subsequently grew MRSA. His blood cultures cleared and on day 9, he underwent a mechanical debulking of the large tricuspid valve vegetations. On day 22, he developed new bilateral pneumothoraces (Figures 3-4).

**Figure 5:**
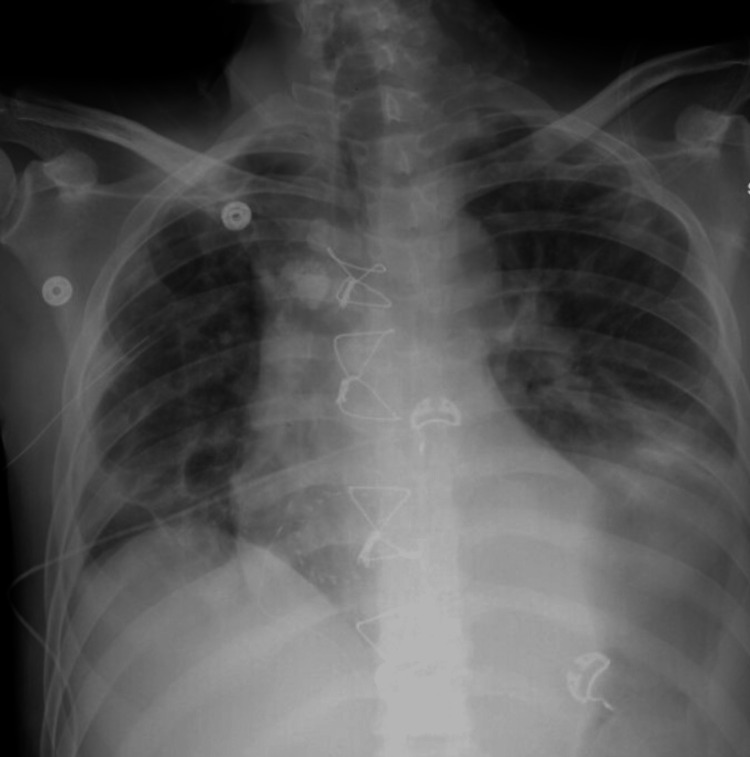
Chest X-ray showing improved pneumothoraces and aeration of both lungs.

By day 52, his clinical status had improved, and a repeat CXR showed improved pneumothoraces and aeration of both lungs (Figure 5).

## Discussion

Pulmonary complications of endocarditis are seen in approximately 7% of patients; however, among patients with IE who are intravenous drug users, pulmonary complications may occur in up to 75%. These complications include pneumonia, lung abscess, pleural effusion, empyema, pulmonary hemorrhage, and rarely pneumothorax [1,3]. Pulmonary abscess formation can lead to the development of large pneumatoceles even after the initiation of appropriate antibiotics. These pneumatoceles can compress adjacent lung parenchyma or cause pneumothorax mimicking the radiographic features of vanishing lung syndrome, which is also referred to as idiopathic giant bullous emphysema [4,5]. If the bullae occupy more than 30% of the hemithorax, they are referred to as giant bullae [6]. The major cause of giant bullae formation is cigarette smoking, and patients typically have a long history of smoking, chronic obstructive pulmonary disease (COPD), history of marijuana use, or alpha-1 antitrypsin deficiency [5,6]. Only one prior case of pneumatocele and vanishing lung syndrome pattern due to endocarditis has been described in the literature [4].

The mechanism of pneumatoceles in IE has not been clearly described in the literature. Hariri et al. used the model by Quigley and Fraser in 1988 to describe the mechanism as the perforations in areas of bronchiolar inflammation leading to air trapping [1]. In addition, the release of proteases from inflammatory cells and a reduction of surfactant production may lead to further airspace enlargement. Finally, septic pulmonary emboli can occlude the pulmonary vascular leading to infarction and necrosis. The bacteria implanted in the lung can stimulate the release of hydrolytic enzymes by the inflammatory cells, leading to liquefactive necrosis. Over the subsequent few weeks, macrophages infiltrate the lung parenchymal to remove the cellular debris leaving a cystic space in the lung. This time period correlated with the onset of pneumatocele and pneumothorax in our patient.

The management of pneumatoceles in patients with IE includes antibiotics, source control with the removal of the vegetation to minimize further embolization to the lungs, and possible chest tube placement for patients with pneumothorax. The pneumatocele and the vanished lung from the IE are usually reversible, as seen in our case.

## Conclusions

IE can be complicated by pneumatocele and vanishing lung syndrome patterns. Our case describes the evolution of pneumatocele and pneumothorax formation as a pulmonary complication of right-sided IE and hypothesized possible mechanisms. It is important to prospectively monitor such patients for these complications. These proactive measures can help improve the health outcomes of patients.
